# Sex differences in circadian food anticipatory activity are not altered by individual manipulations of sex hormones or sex chromosome copy number in mice

**DOI:** 10.1371/journal.pone.0191373

**Published:** 2018-01-31

**Authors:** Antonio Aguayo, Camille S. Martin, Timothy F. Huddy, Maya Ogawa-Okada, Jamie L. Adkins, Andrew D. Steele

**Affiliations:** Department of Biological Sciences, California State Polytechnic University Pomona, Pomona, CA, United States of America; Kent State University, UNITED STATES

## Abstract

Recent studies in mice have demonstrated a sexual dimorphism in circadian entrainment to scheduled feeding. On a time restricted diet, males tend to develop food anticipatory activity (FAA) sooner than females and with a higher amplitude of activity. The underlying cause of this sex difference remains unknown. One study suggests that sex hormones, both androgens and estrogens, modulate food anticipatory activity in mice. Here we present results suggesting that the sex difference in FAA is unrelated to gonadal sex hormones. While a sex difference between males and females in FAA on a timed, calorie restricted diet was observed there were no differences between intact and gonadectomized mice in the onset or magnitude of FAA. To test other sources of the sex difference in circadian entrainment to scheduled feeding, we used sex chromosome copy number mutants, but there was no difference in FAA when comparing XX, XY-, XY-;Sry Tg, and XX;Sry Tg mice, demonstrating that gene dosage of sex chromosomes does not mediate the sex difference in FAA. Next, we masculinized female mice by treating them with 17-beta estradiol during the neonatal period; yet again, we saw no difference in FAA between control and masculinized females. Finally, we observed that there was no longer a sex difference in FAA for older mice, suggesting that the sex difference in FAA is age-dependent. Thus, our study demonstrates that singular manipulations of gonadal hormones, sex chromosomes, or developmental patterning are not able to explain the difference in FAA between young male and female mice.

## Introduction

There is extensive research demonstrating that photic input to the retina entrains the oscillations of neurons in the suprachiasmatic nucleus (SCN) and that these cells, in turn, control a myriad of circadian rhythms [1]. However, there is evidence to suggest that circadian clocks are present in tissues far removed from the SCN that can be entrained by daily cycles of food availability even when the SCN is ablated [2, 3]. In rodents, this rhythm is readily observed by restricting food access (or feeding a calorie restricted meal) to the middle of the light period, when nocturnal rodents normally eat little and are inactive [4], but food anticipatory activity (FAA) can also be detected readily in the night hours or even for multiple meals per day [5]. This induces a pronounced shift in circadian oscillator(s) and peripheral physiology in alignment with the new daily feeding time, while the SCN remains coupled to the light-dark cycle. Additionally, this phenomenon is also associated with the emergence of a burst in locomotor activity that anticipates a scheduled mealtime 1–3 hours prior to feeding [6].

Despite differences in rates of eating disorders amongst human sexes, surprisingly little attention has been paid to sex differences in food entrained rhythms. A sexual dimorphism in FAA could help to indicate the underlying neuronal circuitry behind the food entrained oscillator(s) given that the male and female brain show only a handful of anatomical differences [7]. Recently, evidence for biological sex differences in circadian entrainment to scheduled feeding has emerged [8–10]. In all of these studies, female mice showed a blunted behavioral response to scheduled feeding as compared to males, which showed higher amplitudes of FAA and more rapid entrainment.

Although we were able to replicate a small sex difference in FAA using a timed, 60% calorie restricted (CR) feeding model, our attempts at pinpointing the cause of this sexual dimorphism were unrealized. Determining the gonad- and sex chromosome-independent source responsible for this sex difference in circadian entrainment to scheduled feeding may be useful for identifying the systems and circuitry associated with food entrainment and help uncover the biological reason as to why females are at greater risk of developing eating disorders such as anorexia nervosa.

## Materials and methods

### Animal use, husbandry, and strains

The experiments described below were approved by the Animal Care and Use Committee under protocols 13.025 and 17.006. Mice were acclimated to our facility for at least two weeks before beginning CR. Mice were maintained in static microisolator cages in the following environmental conditions: 12:12 light:dark cycle, temperatures ranged between 21–23°C, and humidity ranged between 45–65%. The cages contained sani-chip bedding (Envigo, product number 7090) and a cotton nestlet. Mice were supplied with dry chow (Irradiated Rodent Diet; Harlan Teklad 2018) and water ad libitum.

**Fig 1:**
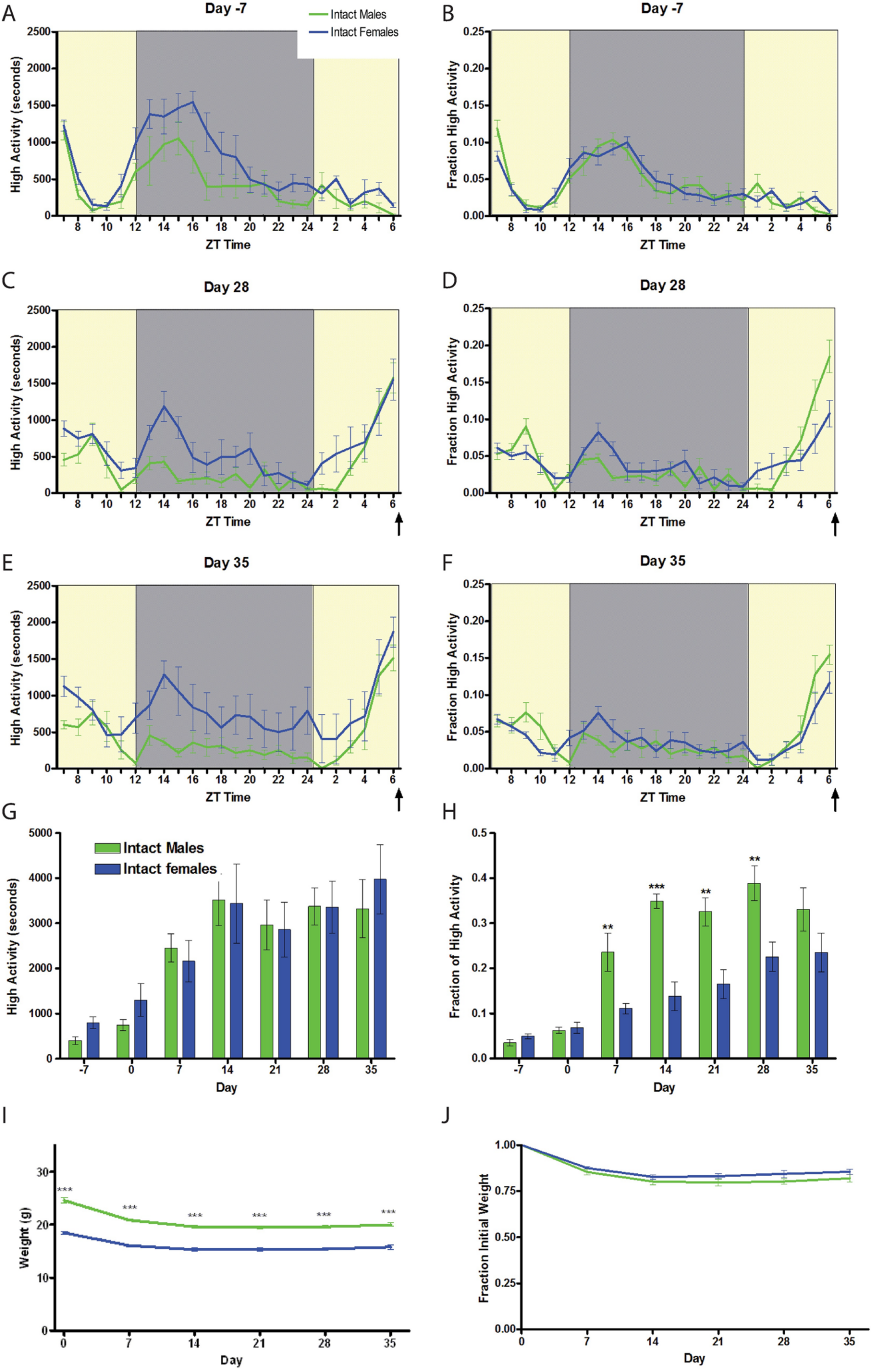
Sex differences in food anticipatory activity. (A) Mean (+/- SEM) seconds of high activity behaviors (hanging, jumping, rearing, and walking) seven days prior to initiating scheduled CR feeding in male (green) and female (blue) mice. (B) Normalized mean high activity of data shown in A. Mean seconds of high activity behaviors after (C) four or (E) five weeks of timed CR feeding. (D,F) Normalized mean +/- SEM high activity of data shown in C and E, respectively. (G) Mean seconds of high activity data in the three hours preceding scheduled feeding across 42 days of the experiment. (H) Mean normalized high activity data in the three hours preceding scheduled feeding across the experiment. (I) Mean body weights for males and female mice on a CR diet for five weeks. (J) Percentage weight loss calculated by normalizing to day 0 weight. n = 8 per group. ** P<0.01; ***P<0.001, Mann-Whitney. The arrow indicates the time of expected food delivery (ZT7).

**Fig 2:**
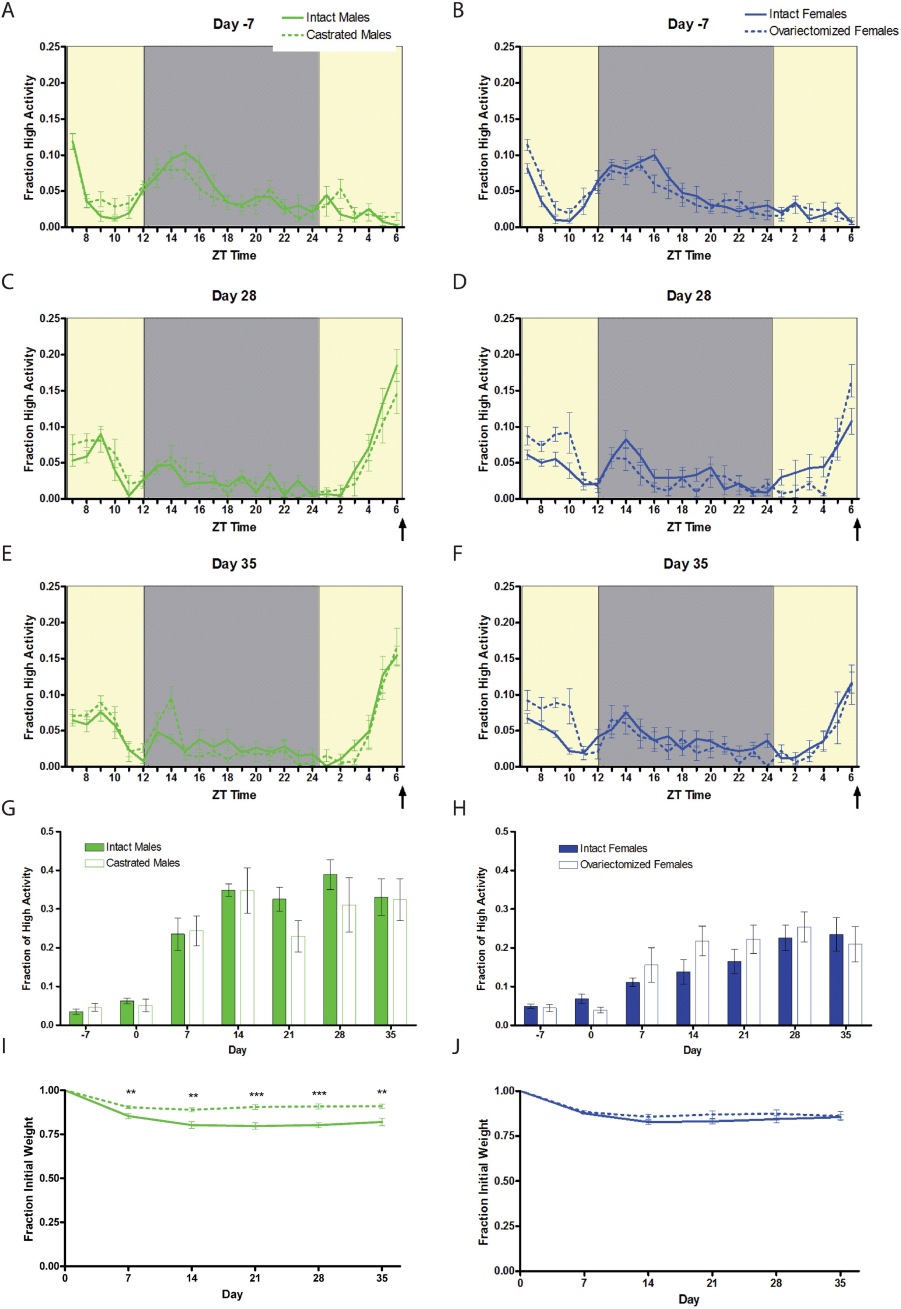
Lack of effect of gonadectomy on food anticipatory activity. Mean (+/- SEM) normalized high activity behaviors seven days prior to initiating scheduled CR feeding in (A) male (solid green) and castrated male (dashed green) and (B) female (solid blue) and ovariectomized (dashed blue) mice. Normalized mean high activity of (C) male and castrated males on day 28, (D) female and ovariectomized females on day 28, (E) male and castrated males on day 35, and (F) female and ovariectomized females on day on day 35. Mean normalized high activity data in the three hours preceding scheduled feeding across 35 days of the experiment for (G) intact versus castrated males and H) intact versus ovariectomized females. (I) Mean body weights loss for intact and castrated males. (J) Mean body weight loss for intact and ovariectomized female mice on a CR diet for five weeks. ** P<0.01; *** P<0.001, Student T test. n = 7–8 per group. The arrow indicated the time of expected food delivery (ZT7).

**Fig 3:**
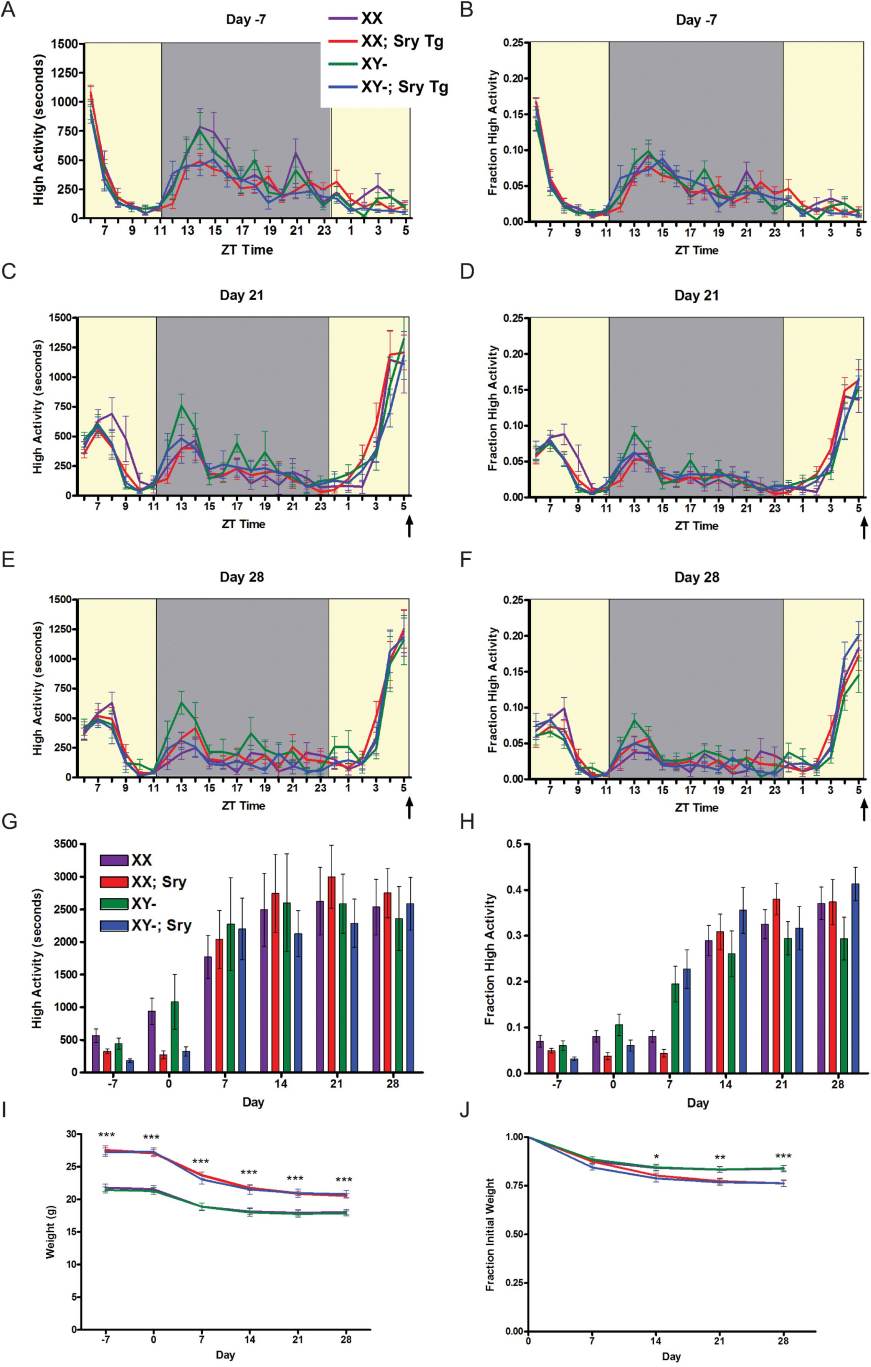
Sex chromosome copy number does not alter food anticipatory activity. (A) Mean (+/- SEM) seconds of high activity behaviors seven days prior to initiating scheduled CR feeding in XX (purple), XX; Sry Tg+ (red), XY- (green), and XY-; Sry Tg+ (blue) mice. (B) Normalized mean high activity of data shown in A. Mean seconds of high activity behaviors after (C) three or (E) four weeks of timed CR feeding. (D,F) Normalized mean high activity of data shown in E and F, respectively. (G) Mean seconds of high activity data in the three hours preceding scheduled feeding across 28 days of the experiment. (H) Mean normalized high activity data in the three hours preceding scheduled mealtime. (I) Mean body weights for males and female mice one week pre-CR and four weeks on a CR diet. (J) Percentage weight loss calculated by normalizing to day 0. * P<0.05, **P<0.01, *** P<0.0001, One-way ANOVA, Tukey-Kramer multiple comparisons. n = 9 for XX mice, n = 12 for XX;Sry Tg mice, n = 11 for XY- mice, and n = 11 for XY-;Sry Tg mice. The arrow indicated the time of expected food delivery (ZT6).

**Fig 4:**
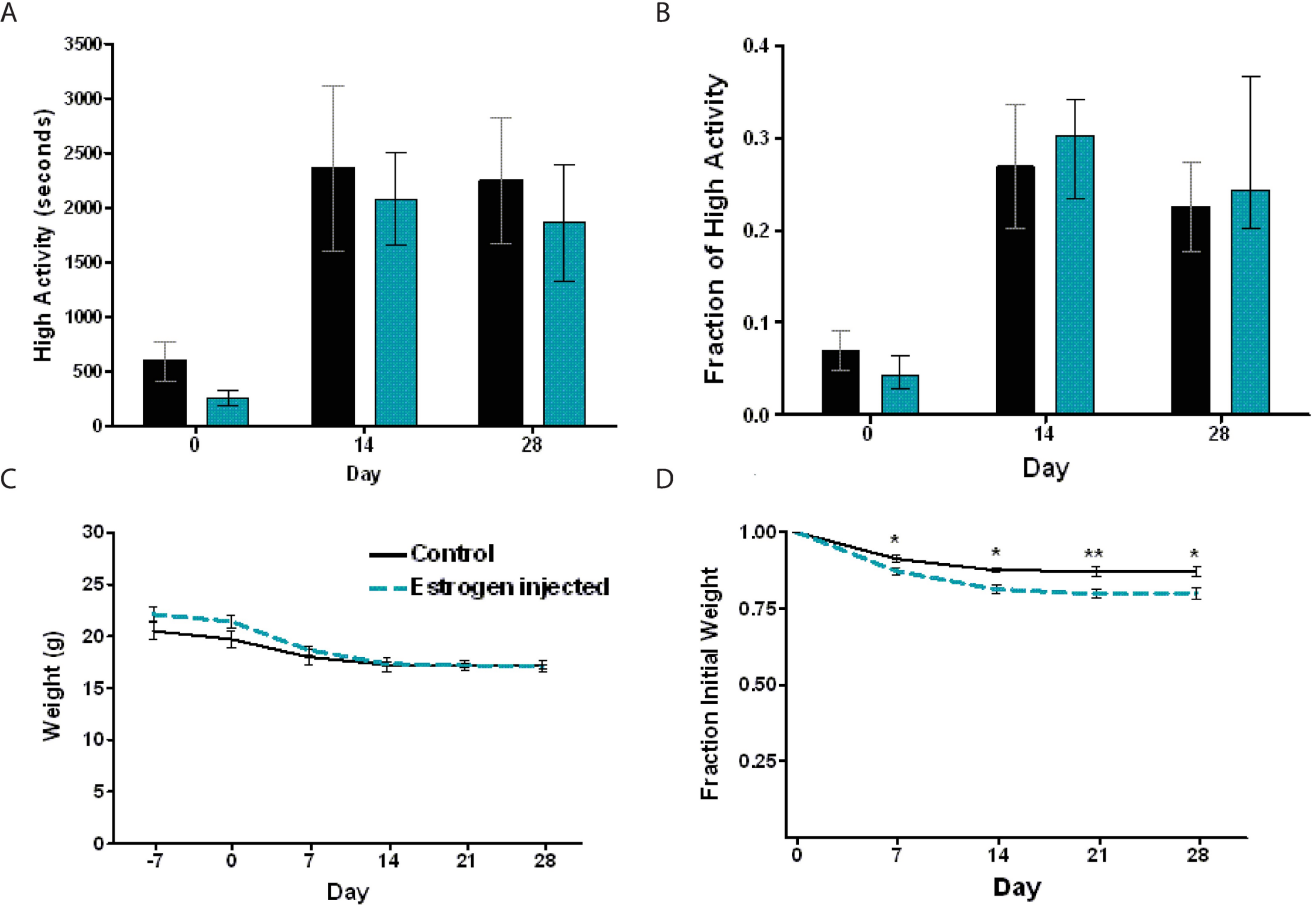
Neonatal 17-beta estradiol brain masculinization does not alter food anticipatory activity. (A) Mean (+/- SEM) seconds of high activity data in the three hours preceding scheduled feeding across 28 days of the experiment. (B) Mean normalized high activity in the three hours preceding scheduled feeding across 28 days of the experiment. (C) Mean body weights for control and 17-beta estradiol -injected mice across the duration of the experiment. (D) Percentage weight loss calculated by normalizing to day 0. * P<0.05; ** P<0.01, Student T test.

**Fig 5:**
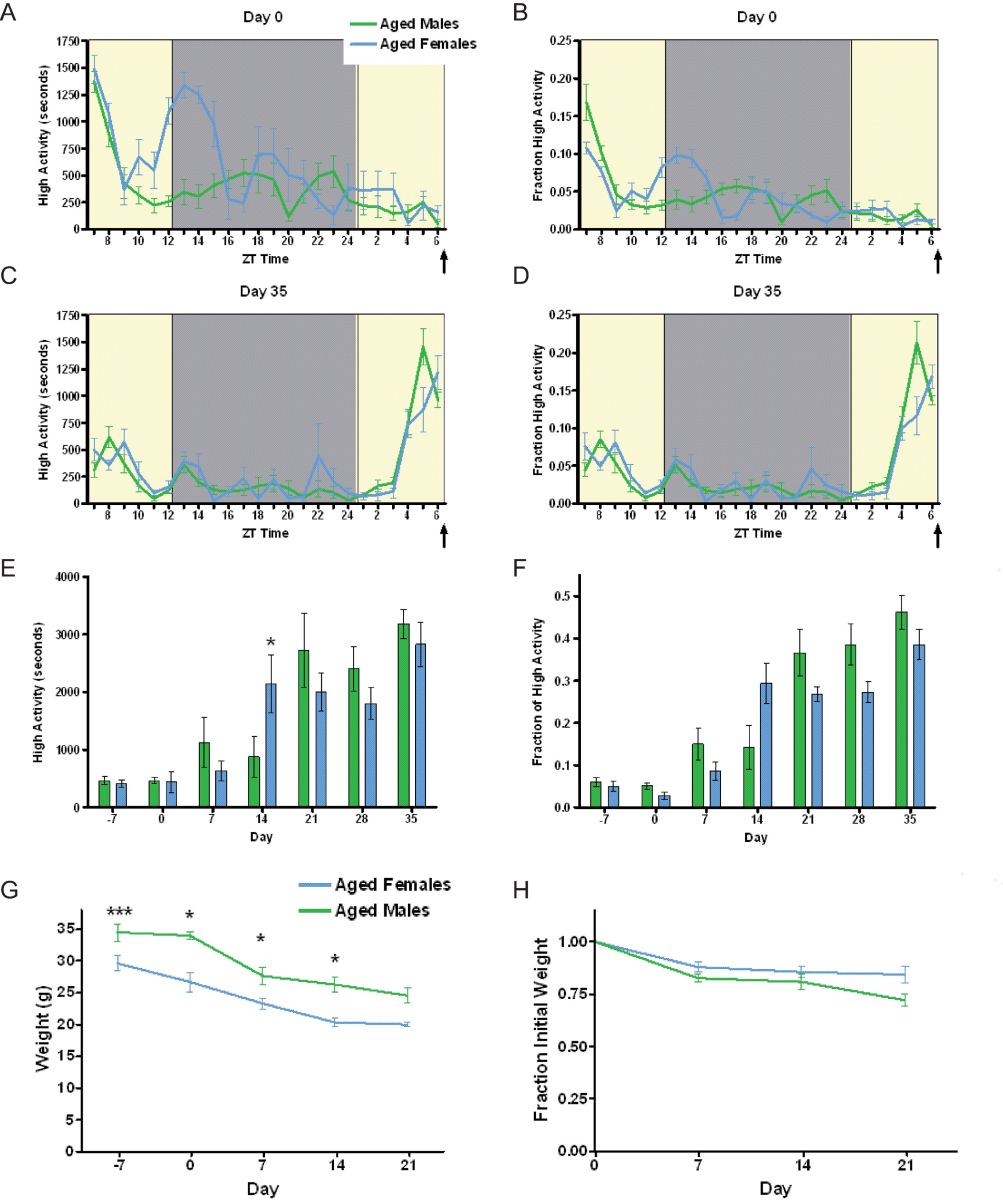
Aged mice do not have a sex difference in food anticipatory activity. (A) Mean (+/- SEM) seconds of high activity behaviors (hanging, jumping, rearing, and walking) on the first day of scheduled CR feeding in male (green) and female (blue) mice. (B) Normalized mean high activity of data shown in A. (C) Mean seconds of high activity behaviors after five weeks of timed CR feeding. (D)Normalized mean high activity of data shown in C. (E) Mean seconds of high activity data in the three hours preceding scheduled feeding across 42 days of the experiment. (F) Mean normalized high activity data in the three hours preceding scheduled feeding across 35 days of the experiment. (G) Mean body weights for aged males and female mice on a CR diet for five weeks. (H) Percentage weight loss calculated by normalizing to day 0. n = 3–6 for females and n = 6–8 for males * P<0.05, ***P<0.001, Mann-Whitney. The arrow indicated the time of expected food delivery (ZT7).

For our initial study of sex differences between male and female mice, we used n = 8 C57BL/6J male and n = 8 females (strain number 000664) purchased from Jackson Labs (Fig 1). For gonadectomy experiments (Fig 2) we used n = 16 male and n = 16 female C57BL/6J mice purchased from Jackson Labs. 8 of the 16 males were castrated and 8 of the 16 females were ovariectomized at 6 weeks of age, and then shipped to our campus where they were acclimated to our facility for two weeks before video recording and measuring of food intake. To study sex chromosome complement (Fig 3) we utilized the “Four Core Genotype (FCG)” model [11]. Mice were obtained from the laboratory of Art Arnold and are also available through Jackson labs as strain 010905. Two male mice with the genotype of XY-; Sry Tg+ were crossed to wild-type C57BL/6J female mice to generate all 43 progeny used for this experiment. We obtained nearly expected ratios of the “4 core genotypes” used for food restriction studies: 9 XX (gonadal female), 11 XY- (gonadal female), 12 XX; Sry Tg (gonadal male), and 11 XY-;Sry Tg (gonadal male) mice. For the brain masculinization experiment (Fig 4), neonatal female C57BL6/J mice (n = 7) were injected three times subcutaneously with 5 micrograms of 17-beta-estradiol (Sigma) dissolved in corn oil at postnatal days 1, 8, and 15 or left untreated as a control (n = 4). For the study of aged C57BL/6 mice (Fig 5), n = 8 males and n = 6 females at 9–10 months of age were used.

To genotype the mice used for the sex chromosome experiment (Fig 3), we extracted genomic DNA from tail clippings. A total of 6 primers were used for genotyping: Myo 1a, Myo b, Ymt R, Ymt F, Sry-R, and Sry-F. The primers with the following sequences were obtained from IDT: Myo 1a (TTACGTCCATCGTGGACAGCAT), Myo b (TGGGCTGGGTGTTAGTCTTAT), Ymt-F (CTGGAGCTCTACAGTGATGA), Ymt-R (CAGTTACCAATCAACACATCAC), Sry-F (AGCCCTACAGCCACATGATA), and Sry-R (GTCTTGCCTGTATGTGATGG). Myo primers served as positive controls for the PCR, Ymt primers identified the Y chromosome, and Sry primers identified the Sry transgene. Multiplexed PCR reactions using DreamTaq Green PCR Master Mix (2x) (ThermoFisher) containing all six primers were carried out but also verified as individual reactions for the Sry transgene.

### Timed calorie restriction studies

For all mice used in the study, food intake was measured across 3–4 days to calculate an average food intake per group. To test for FAA, mice were then allocated 60% of the group average (by mass) daily at ZT 7 (ZT 0 is when the lights turn on) for experiments presented in Figs 1, 2, 4 and 5. For experiments presented in Fig 3 feeding time was at ZT 6. Mice typically consume all of this meal within 1–2 hours once they have been on a CR feeding schedule for one week. All mice were 9–10 weeks old, with the exclusion of the aged mice which were 9 months old, at the time of CR. Body weight measurements were conducted weekly, beginning one week prior to initiating 60% CR (‘day -7’). We attempted to prevent weight loss exceeding 15% by making small adjustments (typically of 0.1g) to the daily food allotment. Thus, the actual “60%” CR value ranged between 60–63% in our studies.

### Behavioral and statistical assessments

Mice were video recorded once per week for 24 hours. Video recordings were analyzed by computer vision software, HomeCageScan 3.0, which quantified home cage behaviors as previously described [12]. For the purposes of our analysis, we only describe activity as rearing, jumping, walking, and hanging for each hour of recording. We define FAA as the amount of high activity behaviors, quantified via HomeCageScan 3.0, occurring in the 3-hours preceding scheduled mealtime divided by the total activity over 24 hours [4, 13, 14].

All p-values were computed using GraphPad Instat and all graphs were made using GraphPad Prism. Graphs display means +/- SEM and sample sizes are indicated both above and in the figure legends. Body weight data, which is normally distributed, was assessed using parametric tests, such as T tests when comparing two groups. Because the home cage behavioral data is often not normally distributed, we used a nonparametric statistical test, such as Mann-Whitney Test when comparing two groups. Specific tests used are indicated in the Results section below. Data are provided in a supplemental file.

## Results

### Food anticipatory activity of young male and female mice

Before initiating CR, we compared the activity of age-matched male and female C57BL/6J mice. We noted that females displayed a trend toward increased high activity behaviors (walking, rearing, jumping, and hanging) during the night (p = 0.09, Mann-Whitney) prior to beginning CR (‘day -7’) (Fig 1A and S1 Table). Subsequently, when on a CR feeding schedule, the females showed significant increases in nocturnal high activity behaviors as compared to males at days 28 and 35 (p = .0006 for day 28 and p = .0003 for day 35, Mann-Whitney) (Fig 1A, 1C and 1E). However, when normalizing the data by dividing the amount of high activity behavior in each hourly bin by the total high activity behavior across 24-hours, the activity waveforms of male and females were superimposable prior to initiating CR (Fig 1B). Males and females showed similar amounts of total time spent engaged in high activity behaviors in the three hours preceding scheduled mealtime when on a 60% CR scheduled diet (Fig 1C, 1E and 1G). However, when we applied our working definition of FAA by the amount of high activity in the three hours prior to scheduled feeding by the total amount of activity, we observed that males had enhanced FAA, demonstrating statistically significant increases in FAA on days 7, 14, 21, and 28 but not day 35 of CR (Fig 1D, 1F and 1H). Thus, although similar absolute levels of FAA were observed (Fig 1G), when accounting for the increased nighttime activity of females a sex difference is apparent. However, this sex difference eventually dissipates with prolonged CR (35 days), suggesting that females may be delayed in the acquisition of FAA but eventually reach a level similar to that of males. The female mice used in our study weighed considerably less than the males (day 0 body weights were 18.5 ± 0.3 and 24.5 ± .5 respectively, p<0.0001, T test; Fig 1I) but both groups showed a similar percentage of body weight loss during CR (p>0.05 at all time points, T test) (Fig 1J).

### Food anticipatory activity in gonadectomized mice

To determine whether higher levels of androgens led to the increased FAA observed in males, we obtained castrated C57BL/6J males and age-matched intact controls. The activity waveforms of castrated and intact males were identical prior to CR (Fig 2A). Both groups showed similar behavioral responses to timed 60% CR (Fig 2C, 2E and 2G). The castrated males, which began our study with much smaller body mass (20.0g ± 0.44) than controls (24.5g ± 0.48), showed only a modest decrease in body weight on 60% CR (Fig 2I). Intact males lost considerably more weight as a percentage of total body weight (p<0.01, T-test, for all measurements after day 0) but this was unsurprising given that their starting body weights were so much higher.

To determine if females showed less FAA than males due to higher levels of estrogens, we obtained ovariectomized C57BL/6J females and age-matched intact female controls. The activity waveforms of ovariectomized and intact females were very similar prior to CR (Fig 2B). Upon a timed CR diet, there were no statistically significant differences in the amount of FAA between ovariectomized and intact females (p>0.05, Mann-Whitney; Fig 2D, 2F and 2H). Both the intact and the ovariectomized females showed similar declines in body weight percentage on CR (Fig 2J).

### Food anticipatory activity in sex reversal y mutant and transgenic mice

To determine if having two X chromosomes was inhibitory for FAA, we utilized the “4 core genotype model” developed by Art Arnold’s laboratory [11]. In this model, mice with a mutation in the *sex reversal y* (*Sry*) gene (denoted at Y-) can be rescued by transgenic expression of *Sry* from an autosome to generate and compare XY-, XX, XY-;Sry Tg, and XX; Sry Tg mice. XY- mice are rendered gonadal females (XY-), whereas XY-;Sry Tg mice are gonadal males. In addition, XX; Sry Tg mice are gonadal males despite having two X chromosomes and can be compared to wild-type females with two copies of the X chromosomes (XX). Prior to CR, all groups had similar amounts of activity (Fig 3A) that was essentially superimposable when normalized by dividing by total activity (Fig 3B). While on CR, the activity levels were similar for all four genotypes and there was a clear induction of FAA at days 21 and 28 of CR (Fig 3C–3F). Both the amount of raw activity and the normalized activity in the 3-hours prior to scheduled meal time were indistinguishable amongst the four genotypes, suggesting that FAA is not influenced by sex chromosome copy number (Fig 3G and 3H). In terms of body weight loss, the XX; Sry and XY-;Sry mice were significantly larger than XY- and XX mice as expected (p<0.0001, One-way ANOVA Tukey-Kramer multiple comparisons test; Fig 3I). Also, somewhat predictably, the percentage of weight loss was more dramatic in groups with larger starting weights (XX;Sry and XY-;Sry mice) (the difference is statistically significant at day 14 p<0.05, day 21 p<0.01, and day 28 p<0.001, One-way ANOVA, Tukey-Kramer multiple comparisons; Fig 3J).

### Food anticipatory activity in neonatal 17-beta estradiol-exposed females

To further probe whether hormonal influences during brain development might account for the lower amount of FAA expressed by female mice, we followed a brain “masculinization” protocol [7]. Female mice were exposed to 17-beta estradiol during the neonatal period at day 1, 8, and 15 or left unexposed as a negative control. In order to test the effectiveness of the 17-beta estradiol-mediated masculinization, we tested the aggressiveness of the treated and untreated female mice in a resident-intruder assay [7]. Five out of seven 17-beta estradiol -injected females showed aggression towards a male intruder mouse whereas only one out of four untreated controls showed aggression towards a male intruder, suggesting that the 17-beta estradiol masculinization protocol was effective. The amount of FAA observed in 17-beta estradiol-treated versus control females were nearly identical after 14 or 28 days of CR (Fig 4A and 4B). The body weights of 17-beta estradiol -injected and control mice were not significantly different prior to or during CR (Fig 4C). However, the 17-beta estradiol -injected females lost more body weight during CR as a percentage of initial weight as compared to control female (Fig 4D). As we did not observe differences in weight loss on CR between male and females (Fig 1H) the significance of this result is unclear. Taken together, our results suggest that brain masculinization by neonatal 17-beta estradiol exposure does not account for the sex difference in FAA observed between male and female mice.

### Food anticipatory activity of aged male and female mice

Finally, we tested middle-aged (9 month old) mice to determine if the sex difference persists with age. Aged females demonstrated an earlier onset of nocturnal activity compared to aged males on Day 0 of the experiment but otherwise had similar activity levels (Fig 5A and 5B). There was a strong induction of FAA in both aged males and females (Fig 5C and 5D). At one point during the experiment, day 14 of CR, aged females showed more FAA in terms of seconds of high activity (p<0.05, Mann-Whitney; Fig 5E) but this difference was not significant when corrected for total activity (Fig 5F). In fact, there were no significant differences in the amount of normalized FAA between aged male and female mice across the entirety of the experiment (p>0.05, Mann-Whitney, Fig 5F). By comparing the normalized FAA data across experiments, we observe that the amount of high activity is similar between young males, aged males, and aged females, which all redistribute about 30–35% of total activity to the 3 hours preceding scheduled mealtime (compare Figs 1J and 5F). The amount of normalized FAA increases in aged females (Fig 5F) compared to young females (Fig 1J), suggesting that the deficit in FAA in females is age-dependent and may be due to developmental mechanisms. Finally, the body weights of aged males were higher than that of aged females, as expected (Fig 5G), but both showed a similar percentage of body weight loss during the first three weeks of CR (data were missing from measurements at later time points).

## Discussion

Sex differences in circadian rhythms were initially described decades ago. For example, Daan and colleagues demonstrated that testosterone was important for maintaining period length and activity amplitude in mice housed in constant darkness [15]. Wheel running activity is well known to vary between the sexes in mice [16] and hamsters [17]. Interestingly, gonadectomy of hamsters did not alter sex differences in light entrainment [17], but did in mice [18]. More recent studies have shown that biological sex may influence light entrained circadian rhythms in a complex manner via developmental, activational, and sex chromosome-dependent mechanisms [19].

With respect to food as a zeitgeber, Dorothy Krieger was the first to note possible sex differences in studies using rats [20]. She observed a trend toward larger temperature shifts in males and a higher amplitude corticosterone rhythm in females in response to scheduled feeding but there were no tests of statistical significance [20]. Scant attention was paid to sex differences in circadian entrainment until very recently. A study from our laboratory of FAA induced by scheduled palatable meals showed that males expressed moderate FAA in response to timed delivery of fatty meals whereas females did not [8]. More recently, sex differences in FAA to time restricted feeding (negative energy balance conditions) were independently confirmed in two laboratories [9, 10]. Michalik and colleagues found an additive effect of deletion of dopamine receptor 1 and female sex on weakening FAA [10]. Li and colleagues utilized gonadectomized mice to address the necessity of sex hormones in mediating sex differences in FAA, observing that androgens promoted while estrogens interfered with FAA [9]. Contrary to the results observed by Li and colleagues, our experiment provides no evidence to suggest that chormones are responsible for a sex difference in FAA.

There are several methodological differences between our study and that of Li et al. that could account for discrepancies observed between our studies. Previously, we have shown that the full spectrum of FAA requires at least three weeks to develop [4, 14]. The time window examined by Li and colleagues was only ten days, which explains why they observe a small fraction of total activity devoted to FAA. For example, in our study we observe that male mice redistributed >30% and females >20% of their total home cage activity to the 3-hours preceding scheduled mealtime (Fig 1H and Fig 2G and 2H). While Li and colleagues reported only about 10% of wheel running activity preceding mealtime as a fraction of total wheel running. In short term studies of FAA, issues such as starting body weight (i.e. fat stores), resistance to weight loss (females are known to be more resistant to weight loss which was observed in Li, et al. study), learning to consume as much food as possible during the temporal feeding window, hunger-induced hyperactivity, and other factors can lead to differences between groups that may not be true circadian phenotypes. A second important methodological difference between studies was the use of home cage activity behaviors versus wheel running. One comparative methods study has suggested that running wheels and telemetric implants yield similar results for food entrainment studies [21], and in the study by Michalik disc running and home cage activity data were quite similar [10]; therefore this methodological difference can be dismissed as a possible cause of discrepant results. A third difference between our study and that of Li et al., was their use of temporal food access (4 hours daily) versus defined caloric restriction (60%) in our study. Previously, we compared time- and calorie-restricted feeding and observed that both methods produce similar amounts of FAA as measured by video-based methods [4], so we do not favor this as an explanation for the discrepancies between our labs. However, given that Li and colleagues observed more substantial weight loss in males than in females and conducted such a short-term study, it is difficult to resolve any of these methodological issues. We attempted to study FAA in males and females using milder CR, restricting to 80% of normal caloric intake but neither males nor females show much FAA under these conditions, similar to what we observed previously in a study of male mice on 60 and 80% CR diets [13].

In summary, we have confirmed that a sex difference in circadian entrainment to scheduled feeding exists, but that the sex difference is not substantial—it is only apparent when normalizing data to total activity—and is absent after prolonged CR (35 days). In fact, older mice (9 months) do not even show a sex difference for FAA. In attempting to determine the mechanism of this small sex difference in young mice, we eliminated gonadal-derived sex hormones in adulthood but found that this did not alter FAA in males or females. We also performed a hormonal manipulation known to masculinize the female brain during early life but this also had no effect on FAA. Vexingly, there was also no difference in FAA between mice varying in sex chromosome copy number: XX females, XY- females, XY-;Sry Tg males, and XX; Sry Tg males. In older mice, there was no difference between the sexes for induction or maintenance of FAA, but it would be interesting to examine much older mice (1.5–2 years). Prior work in rats has shown some deterioration of food rhythms in aged male rats [22]. Taken together, our experiments suggest that combinatorial effects of developmental, hormonal, sex-chromosome related, or other unidentified factor(s) are responsible for the sex difference in circadian entrainment to scheduled feeding. Given known metabolic differences between male and female mice [23, 24], it is not all that surprising to observe small differences in circadian entrainment to scheduled feeding. Finally, the continuing discrepancies between studies of food entrainment underscores the need for standardized experimental protocols and reporting of data [25].

## Supporting information

S1 Table Body weight and behavioral data for all mice shown in the figures. (XLSX)
